# Cerebral tomoelastography based on multifrequency MR elastography in two and three dimensions

**DOI:** 10.3389/fbioe.2022.1056131

**Published:** 2022-12-02

**Authors:** Helge Herthum, Stefan Hetzer, Bernhard Kreft, Heiko Tzschätzsch, Mehrgan Shahryari, Tom Meyer, Steffen Görner, Hennes Neubauer, Jing Guo, Jürgen Braun, Ingolf Sack

**Affiliations:** ^1^ Berlin Center for Advanced Neuroimaging, Charité—Universitätsmedizin Berlin, Corporate Member of Freie Universität Berlin, Berlin Institute of Health, Humboldt-Universität zu Berlin, Berlin, Germany; ^2^ Bernstein Center for Computational Neuroscience, Charité—Universitätsmedizin Berlin, Corporate Member of Freie Universität Berlin, Berlin Institute of Health, Humboldt-Universität zu Berlin, Berlin, Germany; ^3^ Institute of Medical Informatics, Charité—Universitätsmedizin Berlin, Corporate Member of Freie Universität Berlin, Berlin Institute of Health, Humboldt-Universität zu Berlin, Berlin, Germany; ^4^ Department of Radiology, Charité—Universitätsmedizin Berlin, Corporate Member of Freie Universität Berlin, Berlin Institute of Health, Humboldt-Universität zu Berlin, Berlin, Germany

**Keywords:** multifrequency MRE, brain, reproducibility, viscoelasticity, human, *in vivo*

## Abstract

**Purpose:** Magnetic resonance elastography (MRE) generates quantitative maps of the mechanical properties of biological soft tissues. However, published values obtained by brain MRE vary largely and lack detail resolution, due to either true biological effects or technical challenges. We here introduce cerebral tomoelastography in two and three dimensions for improved data consistency and detail resolution while considering aging, brain parenchymal fraction (BPF), systolic blood pressure, and body mass index (BMI).

**Methods:** Multifrequency MRE with 2D- and 3D-tomoelastography postprocessing was applied to the brains of 31 volunteers (age range: 22—61 years) for analyzing the coefficient of variation (CV) and effects of biological factors. Eleven volunteers were rescanned after 1 day and 1 year to determine intraclass correlation coefficient (ICC) and identify possible long-term changes.

**Results:** White matter shear wave speed (SWS) was slightly higher in 2D-MRE (1.28 ± 0.02 m/s) than 3D-MRE (1.22 ± 0.05 m/s, *p* < 0.0001), with less variation after 1 day in 2D (0.33 ± 0.32%) than in 3D (0.96 ± 0.66%, *p* = 0.004), which was also reflected in a slightly lower CV and higher ICC in 2D (1.84%, 0.97 [0.88–0.99]) than in 3D (3.89%, 0.95 [0.76–0.99]). Remarkably, 3D-MRE was sensitive to a decrease in white matter SWS within only 1 year, whereas no change in white matter volume was observed during this follow-up period. Across volunteers, stiffness correlated with age and BPF, but not with blood pressure and BMI.

**Conclusion:** Cerebral tomoelastography provides high-resolution viscoelasticity maps with excellent consistency. Brain MRE in 2D shows less variation across volunteers in shorter scan times than 3D-MRE, while 3D-MRE appears to be more sensitive to subtle biological effects such as aging.

## Introduction

Magnetic resonance elastography (MRE) is an emerging imaging modality which allows *in vivo* assessment of soft tissue mechanics (Jamin et al., 2015; Venkatesh and Ehman, 2015; Murphy et al., 2019). MRE generates quantitative maps of the mechanical properties of biological tissues by stimulating, encoding, and numerically analyzing shear waves. In neuronal applications, MRE has been proven sensitive to disease and physiological effects both for 2D and 3D wave inversion (Hiscox et al., 2016; Hirsch et al., 2017; Yin et al., 2018; Murphy et al., 2019). Prominent examples include brain softening during aging (Sack et al., 2009; Arani et al., 2015; Hiscox et al., 2021) and Alzheimer’s disease (Murphy et al., 2011; Murphy et al., 2016; Hiscox et al., 2020a), multiple sclerosis (Wuerfel et al., 2010; Fehlner et al., 2016), Parkinson’s disease (Lipp et al., 2013; Lipp et al., 2018), and normal pressure hydrocephalus (Streitberger et al., 2011; Freimann et al., 2012). Conversely, brain stiffening has been reported as a result of jugular compression (Hatt et al., 2015), Valsalva maneuver (Herthum et al., 2021a), hypercapnia (Hetzer et al., 2019), perfusion pressure (Hetzer et al., 2018), idiopathic intracranial hypertension (Kreft et al., 2020), and functional activation (Patz et al., 2019; Lan et al., 2020).

Specifically, aging has been reported to be associated with up to 0.8% brain softening per year in adults (Hiscox et al., 2021). Alzheimer’s disease and multiple sclerosis contribute 7% (Murphy et al., 2011; Gerischer et al., 2018) to 20% (Wuerfel et al., 2010; Streitberger et al., 2012; Fehlner et al., 2016) lower brain stiffness while changes in blood perfusion have smaller effects of only 2%–5% (Hetzer et al., 2018; Hetzer et al., 2019). Focal changes such as tumors are delineable by MRE when lesions markedly alter brain stiffness on the order of 100% and, thus, generate robust contrast in viscoelasticity maps (Simon et al., 2013; Bunevicius et al., 2020).

However, possible changes in smaller multiple sclerosis lesions might be masked by blurry or noisy MRE maps, which cannot display interfaces between small anatomical subregions or cerebrospinal fluid (CSF) (Herthum et al., 2022a). For example, deep gray matter (DGM) regions such as the putamen, caudate nucleus, or globus pallidum are still difficult to detect using viscoelasticity maps, which hinders diagnostic applications of brain MRE in those regions (Guo et al., 2013a; Murphy et al., 2013; Hiscox et al., 2020b). Moreover, in disseminated pathologies that affect larger brain regions, MRE is hampered by a relatively wide inter-subject variability of stiffness values. For example, differences in brain stiffness of 13%–20% have been reported between healthy individuals of similar age using the same MRE method (Guo et al., 2013b; Murphy et al., 2013; Dittmann et al., 2016; Hiscox et al., 2020b; Hiscox et al., 2016). It is still unclear whether this variability is due to methodological differences, geometrical reasons such as individual brain morphology, physiological influences including blood pressure (Hetzer et al., 2018; Herthum et al., 2021a) and body mass index (BMI) (Hetzer et al., 2020; Wang et al., 2021), or, if intrinsic structural differences among individuals result in distinct brain stiffness values.

Taken together, we identified two main challenges for the clinical application of state-of-the-art cerebral MRE: first, limited resolution of detail and, second, large inter-subject variability. Since no ground truth values exist for *in vivo* brain stiffness, it remains to be determined if these challenges reflect technical limitations or biological margins of variability of brain viscoelasticity.

Regarding technical challenges, it has been discussed that 3D MRE provides more consistent measurements than 2D MRE because wave patterns in the reverberant skull are rather complex in space and encounter wave guide effects which might disturb planar projections (Romano et al., 2012; Manduca et al., 2018). On the other hand, 2D inversion algorithms are less prone to interslice artifacts and do not require multi-slice acquisitions, which expedites image acquisitions of thinner slabs through the tissue of interest (Tzschatzsch et al., 2016; Mura et al., 2020). To tackle the longstanding question of whether 2D or 3D MRE is preferable for intracranial applications we developed a brain processing pipeline which exploits wavenumber (*k*)-based multifrequency dual elasto-visco (*k*-MDEV) inversion in two variants, once as a new development with shear wave separation in 3D using the curl operator and once with 2D bandpass filtering (Herthum et al., 2022a; Herthum et al., 2022b) [publicly available (Meyer et al., 2022)]. *k*-MDEV supports multifrequency inversion as included in the tomoelastography pipeline that has been used in many multifrequency MRE applications in abdominal and pelvic organs (Dittmann et al., 2017; Shahryari et al., 2019; Reiter et al., 2020). Unlike regional stiffness measurement as performed in standard ultrasound elastography, or when small tissue areas are masked in MRE analysis, tomoelastography provides MRE maps with anatomical detail across the entire field of view. We chose this tomoelastography approach based on *k*-MDEV inversion because it invokes first-order gradients instead of second-order Laplacian operators, making it more robust against noise than direct inversion approaches. Consequently, we expect less noise-related artifact than with previous methods and hope to thus achieve better parameter maps and data consistency.

Since the ground truth for the mechanical properties of *in vivo* brain tissue is unknown, we are left to assess the quality of MRE maps based on the symmetry of the brain as well as anatomical landmarks compared to high-resolution conventional MRI. For example, DGM subregions vary in their relaxation times, providing MRI contrast. Reproducing regional image contrasts of anatomical structures based on shear wave speed would fundamentally change our perception of brain MRE maps as a source of tomographic information beyond regional mean values. Furthermore, we assess consistency by the cross-sectional and longitudinal variation of MRE values across larger anatomical areas [global brain tissue (GBT), cortical gray matter (CGM), white matter (WM), and DGM] as well as the reproducibility of values. Reproducibility is addressed by repeated measurement after a day while longitudinal variation due to possible aging effects is studied by repeated examinations after 1 year.

Regarding the sensitivity of brain MRE to biological effects, we study possible biological influences (cross-sectional age, longitudinal aging, peripheral blood pressure, BMI) and geometrical influences (brain parenchyma fraction, BPF) including wave amplitudes on the measured values by correlation analysis.

Collectively, we aim to

(i) provide reference values for cerebral tomoelastography of the brain,
(ii) demonstrate high-resolution viscoelasticity mapping of anatomical detail,
(iii) assess the short-term and long-term consistency of the method based on 1-day and 1-year follow-up examinations, and
(iv) discuss pros and cons of 2D and 3D wave inversion in MRE of the brain.

The entire data processing pipeline developed in this study is publicly available under https://bioqic-apps.charite.de/(Meyer et al., 2022). We believe that providing reference values and reproducibility scores for different anatomical regions of the human brain, obtained with a processing tool that can be easily accessed by researchers worldwide, will contribute to the urgently needed standardization in MRE.

## Methods

### Volunteers

We included 31 healthy volunteers (12 women; mean age ±standard deviation [SD]: 34 ± 11 years, age range: 22–61 years) in this study. A subgroup of eleven volunteers (3 women; mean age ±SD: 32 ± 9 years, age range: 22–46 years) were examined two additional times, 1 day and 1 year after the baseline examination. All volunteers underwent both standard anatomical MRI and multifrequency MRE.

### Standard anatomical MRI

All experiments were performed in a 3-Tesla MRI scanner (Siemens Lumina, Erlangen, Germany) equipped with a 32-channel head coil. Each volunteer’s head was placed in the same position on the vibration bed with precise connection to the driver during all follow-up examinations. All slice blocks were automatically positioned at the center of the brain using the scanner’s auto align function based on the localizer scan. T1-weighted, high-resolution, whole-brain images were acquired using a magnetization-prepared rapid acquisition of gradient echo sequence (MPRAGE; echo time: 2.27 m, repetition time: 2,300 m, inversion time: 900 m, flip angle of 8°, isotropic voxel size of 1 mm³). WM volume and the brain parenchymal fraction (BPF), which is the ratio of intracranial brain parenchymal volume (GM plus WM) to total intracranial volume (GM plus WM plus CSF) were calculated from MPRAGE images using the segmentation routine *SPM-segment* from the neuroimaging data analysis package SPM12 (Penny et al., 2011).

### MRE experimental setup

**FIGURE 1:**
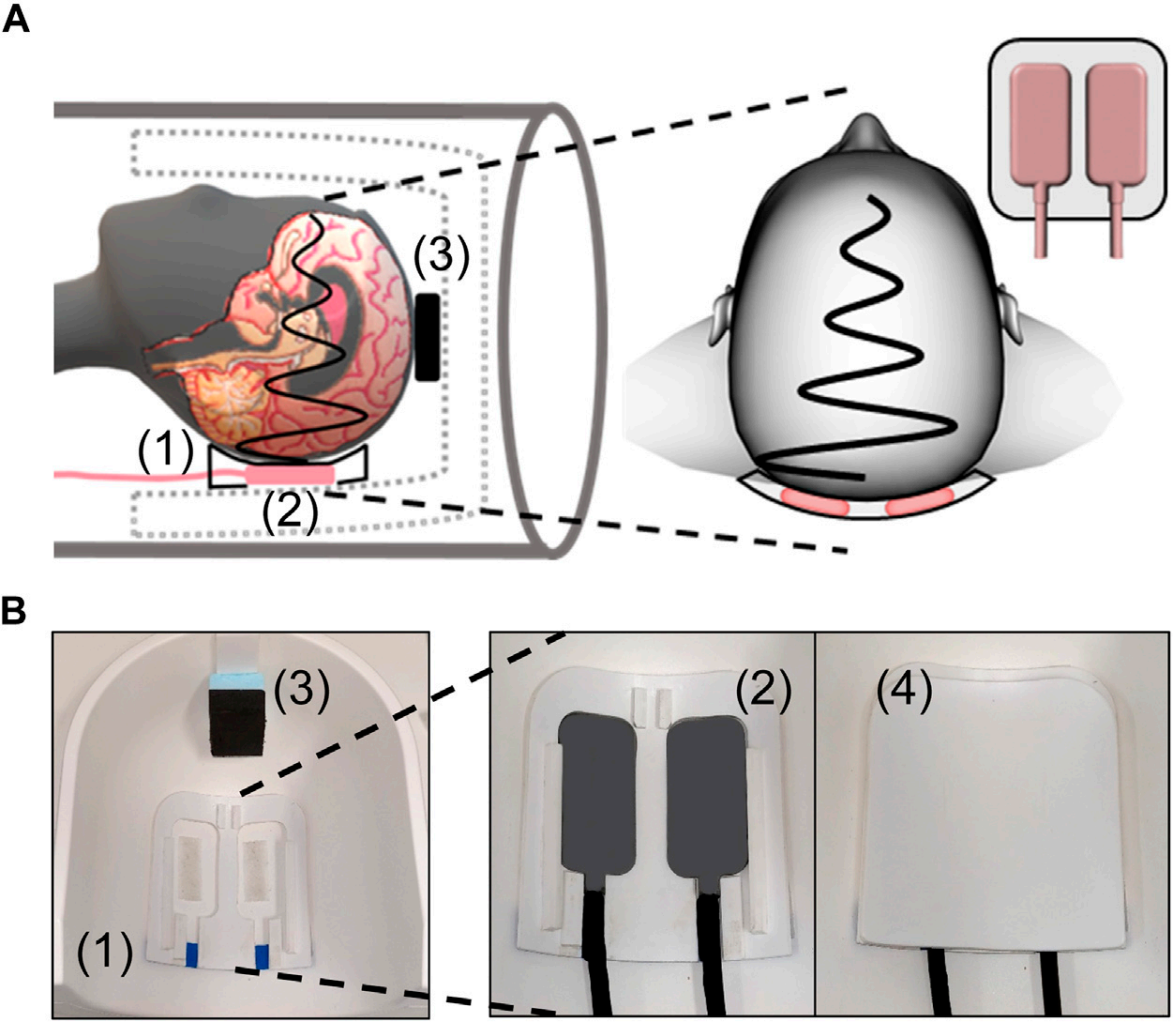
MRE experimental setup. **(A)** Schematic of positioning the head within the head coil with a placeholder (1) for two actuators (2) underneath the head and a spacer (3) at the top of the head. The close view shows the lateral positioning of the two actuators inside the placeholder. **(B)** Photographs of placeholder (1) and spacer (3) in the head coil while actuators (2) inside the placeholder and the transmission plate (4) covering the actuators are shown in close-up.

Multifrequency MRE was performed using a single-shot, spin-echo, echo-planar imaging (EPI) sequence (Dittmann et al., 2016). Eight phase offsets equally spaced over a vibration period were recorded for 40 axial slices for each harmonic vibration induced at 20, 25, 30, and 35 Hz using a pressurized air driver. The driver consists of two tubes connected to custom-made, 3D printed air cushions which are covered by a transmission plate. The cushions were held in place and positioned using a placeholder underneath the subject’s head inside the head coil. A detailed setup is displayed in Figure 1. The driver was operated in opposed-phase mode to reduce bulk motion in anterior-posterior direction. Vibrations started 3 s before data acquisition to ensure harmonic motion without transient effects. Three displacement components in orthogonal directions were encoded using a flow-compensated, motion-encoding gradient with an amplitude of 34 mT/m and a duration of 28 m. Encoding efficiencies were 12.4, 8.9, 7.1, and 6.2 μm/rad for 20, 25, 30, and 35 Hz, respectively. Further imaging parameters were: field of view 202 × 202 mm^2^, voxel size 1.6 × 1.6 × 2 mm^3^, acquisition matrix 126 × 126 × 40, echo time 70 m, repetition time 4,700 m. GRAPPA parallel acquisition (Griswold et al., 2002) with an acceleration factor of two was used. Moreover, two images with inverted phase-encoding direction were recorded for distortion correction. Total acquisition time for a full set of 3D multifrequency MRE data including 40 slices and four frequencies sampled with eight timesteps in three directions was approximately 8 min.

Complex MRE images were corrected in a slice-wise fashion (2D) for stochastic head motion and field distortions using *SPM-realign* and *Hysco2* (based on field maps with inverted readout direction), respectively, in SPM12. Mean MRE magnitude images were calculated by averaging over the frequencies, encoding directions, and time steps and normalized to the Montreal Neurological Institute (MNI) space based on the ICBM152 template (Mazziotta et al., 2001) using SPM12. Generated transformation matrices were used for normalization of viscoelastic parameter maps.

### MRE data analysis

Shear wave speed (SWS, in m/s) and penetration rate (PR, inverse wave attenuation in m/s) maps (Manduca et al., 2021) were reconstructed using wavenumber-based (*k*-)MDEV inversion (Tzschatzsch et al., 2016) with recently introduced, brain-adapted pre-processing (Herthum et al., 2021b; Herthum et al., 2021c; Herthum et al., 2022a). The equation of motion for time-harmonic shear waves in MRE is the wave equation for shear deformations in the linear-elastic range. MRE displacement amplitudes give rise to maximum shear strain amplitudes in the range of 0.1%, which is well within the linear elastic range (Hirsch et al., 2013). *k*-MDEV assumes plane shear waves $u\left(r,\;t\right)$ with complex wave number $k^{*}$ ( $k^{*}=k^{\prime }+ik^{\prime \prime }$ ), angular frequency *ω* and amplitude ***u***_***0***_ as solutions of the governing wave equation:

$$u\left(r,\;t\right)=u_{0}∙e^{i\left(k^{*}∙r-\omega t\right)}$$ (1)

Therefore, as a critical step of preprocessing, *k*-MDEV decomposes the measured vector fields $u\left(r,\;t\right)$ after temporal Fourier transformation into complex-valued scalar wave fields ${\tilde{u}}_{dcf}$ for each propagation direction (*d*), wave component (*c*) and frequency (*f*). Wave numbers are then deduced from the phase gradient of $u_{dcf}$ :

$$k_{dcf}^{\prime }=\left\|\nabla \arg\left(u_{dcf}\right)\right\|$$ (2a)

$$k_{dcf}^{\prime \prime }=\left\|\frac{\nabla \left|u_{dcf}\right|}{\left|u_{dcf}\right|}\right\|$$ (2b)

Compound property maps of SWS and PR are finally obtained by weighted-averaging (weights w) over components, directions, and N frequencies:

$${SWS}_{f}=\frac{\omega \sum_{d}\sum_{c}w}{\underset{d}{\sum }\underset{c}{\sum }k_{dcf}^{\prime }w}\;,\;with\;w={\left|u_{dcf}\right|}^{2}$$ (3a)

$$SWS=\frac{N}{\overset{N}{\underset{f=1}{\sum }}\frac{1}{{SWS}_{f}}}$$ (3b)

$${\mathrm{PR}}_{f}=\frac{\omega }{2\pi }\frac{\sum_{d}\sum_{c}w}{\sum_{d}\sum_{c}k_{dcf}^{\prime \prime }w},\;with\;w={\left|u_{dcf}\right|}^{2}$$ (3c)

$$\mathrm{PR}=\frac{N}{\overset{N}{\underset{f=1}{\sum }}\frac{1}{{\mathrm{PR}}_{f}}}$$ (3d)

SWS and PR are related to the well-established complex shear modulus G* with its real part or storage modulus *G′* and its imaginary part or loss modulus *G″*, with density ρ = 1,000 kg/m^3^:

$$SWS=\sqrt{\frac{2\left({G^{\prime }}^{2}+{G^{\prime \prime }}^{2}\right)}{\rho \left(\sqrt{{G^{\prime }}^{2}+{G^{\prime \prime }}^{2}}+G^{\prime }\right)}}$$ (4a)

$$\mathrm{PR}=\frac{1}{2\pi }\sqrt{\frac{2\left({G^{\prime }}^{2}+{G^{\prime \prime }}^{2}\right)}{\rho \left(\sqrt{{G^{\prime }}^{2}+{G^{\prime \prime }}^{2}}-G^{\prime }\right)}}$$ (4b)

No further assumptions or conversions to other viscoelastic parameters were utilized. SWS is related to tissue stiffness and will be termed as such where appropriate. PR reflects inverse attenuation, i.e., the deeper the shear waves penetrate the tissue the less viscous the tissue behaves and is therefore related to viscosity. Reconstructions were performed 2D (slice-wise) and fully 3D using a newly developed processing pipeline. For 2D data processing, wave images were decomposed in eight propagation directions. Smoothing and suppression of compression waves were done using a bandpass Butterworth filter of third order with a highpass threshold of 15 1/m and lowpass threshold of 250 1/m. SWS and PR maps were reconstructed based on 2D phase gradients. The 2D pipeline is publicly available on https://bioqic-apps.charite.de/ (Meyer et al., 2022). For 3D data processing, slice phase offsets and interphase discontinuities between slices were removed after temporal Fourier transform according to Barnhill et al. (2019). The corrected images were smoothed with a lowpass Butterworth filter of first order and threshold of 200 1/m. Single-direction shear wave fields were computed using the 3D curl operator with 3-pixel symmetric derivative kernels followed by spatial filtering into 20 directions equally distributed over a 3D sphere. SWS and PR maps were reconstructed based on 3D phase gradients. According to Eqs 3b, 3d, wave numbers of all frequencies, wave components and wave propagation directions were averaged without further consideration of wave dispersion to stabilize the inversion, as originally proposed in (Tzschatzsch et al., 2016).

Due to edge slice artifacts of the 3D inversion, the four outermost slices in each direction were removed, leaving 32 slices for further analysis. Edge slice artifacts from finite difference operators are common for 3D MRE (Murphy et al., 2013) but were slightly enlarged in our implementation by the directional filter. The same number of slices was discarded from our 2D analysis to ensure comparability of values obtained in the same volumes.

SWS and PR maps were normalized to the MNI space using the mean MRE magnitude images to generate averaged parameter maps and tissue probability maps (Penny et al., 2011). Probability maps for WM, CGM, and DGM were thresholded at 0.5 to generate segmentation masks. Probabilities for cerebrospinal fluid were thresholded at 0.1 and excluded from other masks to avoid tissue-fluid boundary artifacts. Spatially averaged values were determined in the following regions: GBT, WM, CGM, and DGM as well as DGM subregions: nucleus accumbens (Ac), nucleus caudate (Ca), globus pallidus (Pal), putamen (Pu), and thalamus (Th). The hippocampus and amygdala were not included since both regions were only covered partially due to their basal positions within the scan volume. In addition, average wave amplitudes were determined in the respective brain regions.

### Dependence of 3D SWS values on number of slices

In eleven volunteers, we further analyzed how the number of slices for a fixed slice block thickness potentially affected SWS and PR values in 3D processing. Starting with 3D processing based on 39 slices, equivalent to 62.4 mm block thickness, we averaged SWS and PR within WM visible in the center slice for reference, and, subsequently, removed the two outermost slices from further 3D processing. The error (in %) relative to the central reference slice was averaged over all volunteers. Data were processed in MATLAB 2020a (Mathworks Inc. Natick, MN, United States).

### Statistical analysis

Cross-sectional investigations provided inter-subject variability based on the coefficient of variation (CV) in all brain regions we analyzed. Correlations between reconstructed parameters (2D-SWS, 2D-PR, 3D-SWS, and 3D-PR) and region-specific wave amplitude, age, systolic peripheral blood pressure (BP), BMI, and BPF were analyzed in GBT using Pearson’s correlation coefficient. *p*-values were corrected for four comparisons using the Holm-Bonferroni method. A multivariable linear regression model with age and BPF as independent variable was calculated for SWS and PR, respectively (e.g., SWS = intercept + beta1*BPF + beta2*age).

Differences between viscoelastic values reconstructed from 2D and 3D inversion and between different brain regions (WM vs. CGM and WM vs. DGM) were analyzed using a paired Student’s t-test and a correlation analysis. *p*-values were corrected for multiple comparisons using the Holm-Bonferroni method.

Repeated measurements in eleven volunteers after 1 day provided reproducibility indices for 2D and 3D data processing based on mean relative absolute difference (RAD) and intraclass correlation coefficient (ICC) for GBT, WM, CGM, and DGM. RAD between 2D and 3D data processing was compared using a paired Student’s t-test. ICC estimates and their 95% confident intervals were based on a single-rater, absolute-agreement, two-way, mixed-effects model (Bédard et al., 2000; Bland and Altman, 2003; Everitt and Howell, 2021). One-year follow-up measurements were compared with earlier measurements in the same volunteer to test for a possible aging effect on values in the GBT, WM, CGM, and DGM. Therefore, we performed two separate paired Student’s t-tests for each region. *p*-values were corrected for two comparisons using the Holm-Bonferroni method.

All statistical analysis was done in R version 4.0.2 (R-Foundation, Vienna, Austria). *p*-values below 0.05 were considered statistically significant.

## Results

The analyzed MRE volumes covered 65% GBT, 77% WM, 53% CGM, and 84% DGM of the MNI volume, resulting in group-averaged volumes of 909 ± 44 cm^3^, 544 ± 21 cm^3^, 380 ± 22 cm^3^, and 53 ± 5 cm^3^, respectively. Mean BPF was 0.77 ± 0.04%.

**FIGURE 2:**
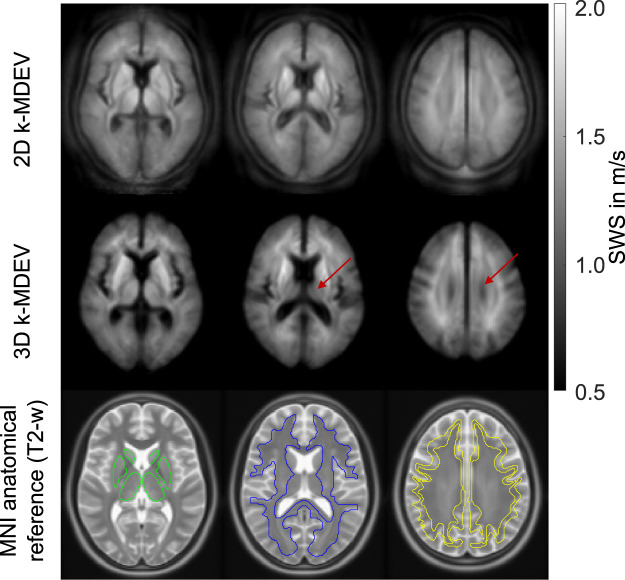
Averaged SWS maps from 2D and 3D *k*-MDEV inversions normalized to MNI space in three representative slices. Red arrow indicates where boundary artifacts from adjacent slices become visible in 3D reconstruction. Anatomical reference images (ICBM152 template) are shown superimposed with atlas regions for deep gray matter (green), white matter (blue), and cortical gray matter (yellow).

**FIGURE 3:**
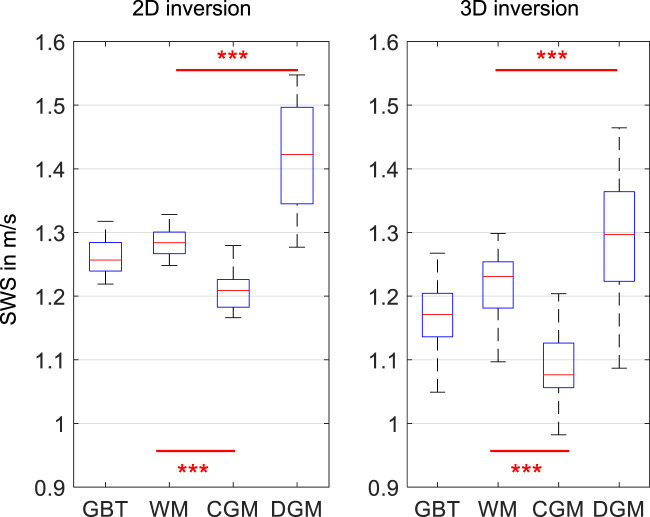
Group mean SWS values for 2D and 3D *k*-MDEV for global brain tissue (GBT), white matter (WM), cortical gray matter (CGM), and deep gray matter (DGM). Significance levels, indicated by asterisks, were determined from paired t-tests with Holm-Bonferroni correction between WM and CGM as well as WM and DGM.

**FIGURE 4:**
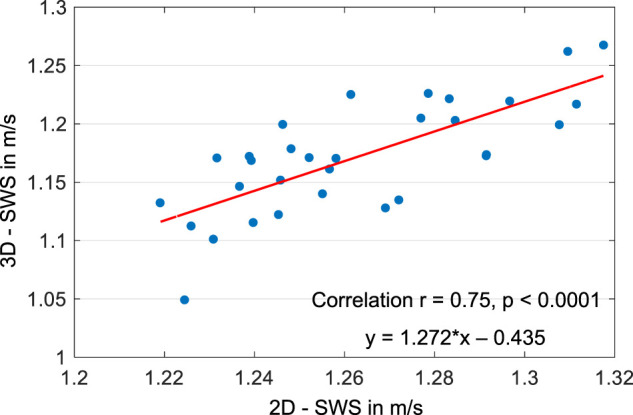
Correlation plot for 2D and 3D SWS values for global brain tissue.

Figure 2 shows three representative slices in MNI space of group-averaged SWS generated by 2D and 3D processing along with anatomical reference images from the MNI atlas. Masks for WM, CGM, and DGM, after the exclusion of cerebrospinal fluid, are demarcated by colored lines while red arrows indicate where 3D boundary artifacts propagated through the slices. Both approaches resulted in high-resolution SWS maps with details of anatomy which visually matched the anatomical reference images. Tissue boundaries were well defined and DGM regions could be visually differentiated from WM based on SWS. Fluid-filled spaces appeared larger in 3D SWS than 2D SWS maps, because the curl operator implied in 3D processing enhanced boundary effects by spatial derivatives (Lilaj et al., 2021), while noise in air was better suppressed by 3D than 2D processing. Mean WM SWS values were slightly higher for 2D (1.28 ± 0.02 m/s) than 3D processing (1.22 ± 0.05 m/s, *p* < 0.0001). Both reconstruction methods yielded higher SWS values for DGM (2D: 1.42 ± 0.09 m/s, 3D: 1.29 ± 0.09 m/s) and lower SWS values for CGM (2D: 1.21 ± 0.03 m/s, 3D: 1.09 ± 0.05 m/s) compared with WM (*p* < 0.0001 for each test). Group statistical plots for GBT, WM, CGM, and DGM in 2D and 3D are shown in Figure 3. A descriptive summary for all analyzed regions is given in Table 1, including region size and CV. Inter-subject variations as quantified by CV were smaller in 2D than 3D processing. CV in GBT, WM, CGM, and DGM was 2.3%, 1.8%, 2.5%, and 6.1% in 2D MRE versus 4.1%, 3.9%, 4.7%, and 6.7% in 3D MRE, respectively (see also Figure 7 and Table 3). Figure 4 shows a correlation plot for 2D and 3D SWS values for GBT. The results for both approaches were highly correlated (*r* = 0.75, *p* < 0.0001). A corresponding analysis is provided for the viscosity-related PR parameter in the Supplementary Material. In short, 2D-PR was markedly higher than 3D-PR (e.g., for GBT: 0.83 ± 0.04 m/s vs. 0.56 ± 0.03). CVs were higher than for SWS and similar between 2D and 3D (e.g., for GBT: 5.4% for 2D and 4.4% for 3D).

**TABLE 1:** Group mean values of SWS for 2D and 3D data processing and the coefficient of variation (CV) for all analyzed brain regions obtained in 31 brains (cross-sectional study): global brain tissue (GBT), white matter (WM), cortical gray matter (CGM), deep gray matter (DGM), nucleus accumbens (Ac), nucleus caudate (Ca), globus pallidus (Pal), putamen (Pu), and thalamus (Th). Standard deviations are given in brackets. In addition, region size is given.

	2D-SWS in m/s	CV in %	3D-SWS in m/s	CV in %	Size in cm^3^
GBT	1.26 (0.03)	2.3	1.17 (0.05)	4.1	909 (44)
WM	1.28 (0.02)	1.8	1.22 (0.05)	3.9	544 (21)
CGM	1.21 (0.03)	2.5	1.09 (0.05)	4.7	379 (22)
DGM	1.42 (0.09)	6.1	1.29 (0.09)	6.7	53 (5)
Ac	1.33 (0.12)	8.7	1.24 (0.10)	7.8	1.6 (0.4)
Ca	1.37 (0.19)	13.5	1.21 (0.19)	16.1	10.1 (0.1)
Pal	1.36 (0.12)	9.1	1.24 (0.11)	8.5	5.5 (0.8)
Pu	1.45 (0.07)	4.7	1.45 (0.08)	5.7	16.5 (1.7)
Th	1.36 (0.10)	7.4	1.11 (0.10)	9.1	26.3 (2.0)

### Correlation analysis

SWS in GBT was negatively correlated with age (2D: *r* = −0.54, *p* = 0.007, 3D: GBT: *r* = −0.45, *p* = 0.04) and positively correlated with BPF (2D: *r* = 0.72, *p* < 0.0001, 3D: GBT: *r* = 0.68, *p* = 0.0001). The annual change in SWS of GBT was -0.0014 m/s in 2D (95%-CI: [−0.0022, −0.0006]) and −0.0019 m/s in 3D (95%-CI: [−0.0034, −0.0005]). Multivariable analysis for SWS in GBT showed a significant effect of BPF on SWS (beta1 = 0.49, standard error = 0.14, *p* = 0.001) while no significant effect of age was observed (beta2 = -0.00027, standard error = 0.0004, *p* = 0.54) given an intercept of 0.90 (standard error = 0.11).

2D SWS of DGM was correlated with wave amplitudes in DGM (mean ± SD: 10.8 ± 2.5 μm, range: 6.4—15.9 μm, *r* = 0.45, *p* = 0.035, slope: 0.015 m/s/μm) while no such correlation was observed in 3D. SWS was not correlated with BP or BMI. A correlation analysis of PR, presented in the Supplementary Material, showed that PR correlated most markedly with wave amplitude in 2D and 3D and only slightly with BPF in 3D.

### Repeated SWS measurement

**FIGURE 5:**
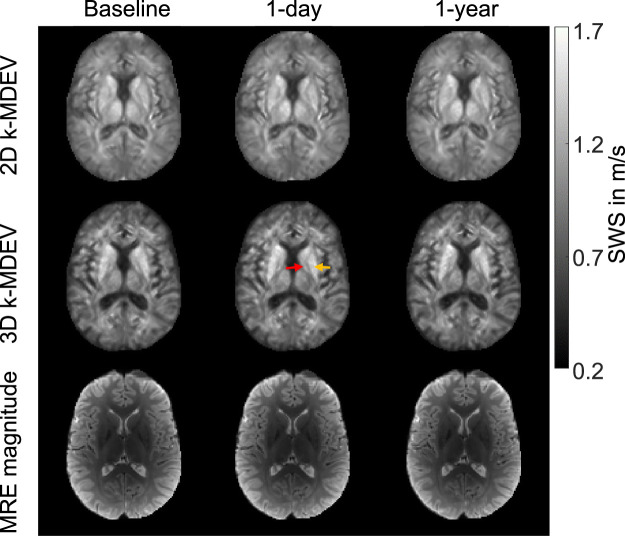
Representative MRE stiffness maps and magnitude images from one volunteer examined at three time points: baseline, 1 day later (1-day), and 1 year later (1-year) for 2D (top) and 3D *k*-MDEV-based reconstruction (bottom). The red arrow points to globus pallidus while the yellow arrow points to putamen. Both regions are clearly distinguishable by eye, consistent with distinct group-averaged values.

Figure 5 shows the reconstructed SWS maps in a representative slice from one volunteer examined at three time points: baseline, 1 day later, and after 1 year for both 2D and 3D processing. No differences between the three measurements were visually apparent. Remarkably, subtle differences between DGM subregions were already apparent based on individual SWS maps and were consistent with group mean values (Figure 2 and Table 1). For example, putamen (yellow arrow) appeared as the stiffest DGM region - in agreement with the results compiled in Table 1 and published values (Hetzer et al., 2018)—and was even distinguishable by eye from globus pallidus (red arrow) in the 3D SWS maps shown in the figure, again in agreement to group mean values (17% difference, *p* < 0.0001). Again, fluid-filled spaces were slightly enlarged by 3D data processing, however, with lower noise than visible in 2D SWS maps.

**FIGURE 6:**
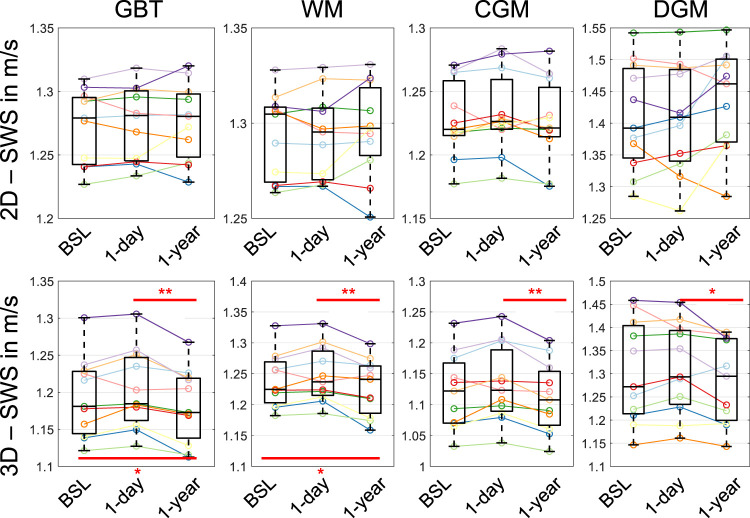
Group-averaged SWS values for 2D (top) and 3D processing (bottom) in global brain tissue (GBT), white matter (WM), cortical gray matter (CGM), and deep gray matter (DGM). Averages were derived from eleven volunteers examined at baseline (BSL), 1 day later (1-day), and after 1 year (1-year). Significance levels, indicated by asterisks, were determined from paired t-tests with Holm-Bonferroni correction between BSL and 1-year as well as 1-day and 1-year.

**FIGURE 7:**
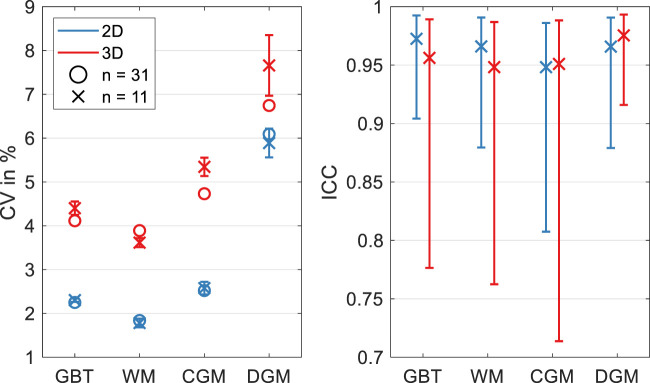
Coefficient of variation (CV, left) and intraclass correlation coefficient (ICC, right) for 2D and 3D SWS reconstructions for global brain tissue (GBT), white matter (WM), cortical gray matter (CGM), and deep gray matter (DGM). CV determined from single examination of all volunteers and as an average from three CVs in eleven volunteers examined at baseline, 1 day later, and after 1 year. ICC was determined from baseline and repeated examination after 1 day.

Group-averaged SWS values (2D and 3D for GBT, WM, CGM, and DGM) measured at three time points in eleven volunteers are presented in Figure 6. Inter-subject variability, assessed by CV, as well as reproducibility between baseline and retest 1 day later, assessed by ICC and mean RAD (within-subject variability), were derived from these results and are displayed in Figure 7. Tables 2, 3 summarize the 1-day test-retest and 1-year follow-up results, respectively.

**TABLE 2:** Coefficient of variation (CV) and intraclass correlation coefficient (ICC) for 2D and 3D SWS reconstructions for global brain tissue (GBT), white matter (WM), cortical gray matter (CGM), deep gray matter (DGM) and DGM subregions. CV is given as an average of three CVs for baseline, 1-day, and 1-year measurement in eleven volunteers (*n* = 11). ICC and mean relative absolute difference (RAD) were determined from baseline and 1-day repeat measurement.

2D SWS	Mean CV (SD), *n* = 11	ICC (95%-CI: Low, up)	Mean RAD (SD, max) in %
GBT	2.30 (0.04)	0.97 (0.90, 0.99)	0.43 (0.33, 1.04)
WM	1.78 (0.10)	0.97 (0.88, 0.99)	0.33 (0.32, 0.91)
CGM	2.58 (0.14)	0.95 (0.81, 0.99)	0.68 (0.46, 1.51)
DGM	5.89 (0.33)	0.97 (0.88, 0.99)	1.31 (1.06, 3.78)
Ac	7.21 (0.31)	0.98 (0.94, 1)	1.12 (1.12, 0.73)
Ca	14.41 (0.36)	0.99 (0.95, 1)	2.29 (2.29, 1.01)
Pal	8.3 (1.19)	0.96 (0.86, 0.99)	2.18 (2.18, 1.48)
Pu	4.2 (0.17)	0.96 (0.87, 0.99)	1.04 (1.04, 0.59)
Th	8.1 (0.8)	0.97 (0.88, 0.99)	1.59 (1.59, 1.81)
**3D SWS**	**Mean CV (SD), *n* = 11**	**ICC (95%-CI: Low, up)**	**Mean RAD (SD, max) in %**
GBT	5.89 (0.33)	0.96 (0.78, 0.99)	1.16 (0.71, 2.25)
WM	3.62 (0.10)	0.95 (0.76, 0.99)	0.96 (0.66, 1.80)
CGM	5.35 (0.21)	0.95 (0.71, 0.99)	1.47 (0.98, 3.49)
DGM	7.66 (0.69)	0.98 (0.92, 0.99)	1.34 (1.17, 3.57)
Ac	6.93 (0.47)	0.92 (0.72, 0.98)	2.48 (2.48, 1.62)
Ca	18.84 (0.63)	0.99 (0.97, 1)	2.04 (2.04, 1.85)
Pal	10.3 (2.06)	0.94 (0.81, 0.98)	3.38 (3.38, 2.34)
Pu	6.04 (0.21)	0.97 (0.88, 0.99)	1.43 (1.43, 0.71)
Th	10.26 (0.99)	0.96 (0.88, 0.99)	2.08 (2.08, 1.8)

**TABLE 3:** SWS results of 1-year follow-up examination compared to the baseline and 1-day measurement as references for 3D data processing. Absolute changes in SWS with 95% confidence intervals and Holm-Bonferroni corrected *p*-values are given for global brain tissue, white matter, cortical gray matter, and deep gray matter.

Region	Parameter	BSL vs. 1-year	1-day vs. 1-year
Global brain tissue	∆SWS in m/s	−0.011	−0.021
	95%—CI (low, up)	(−0.020, −0.002)	(−0.030, −0.011)
	*p*-value	0.0263	0.0014
White matter	∆SWS in m/s	−0.011	−0.019
	95%—CI (low, up)	(−0.020, −0.001)	(−0.030, −0.009)
	*p*-value	0.0367	0.0068
Cortical gray matter	∆SWS in m/s	0.009	−0.022
	95%—CI (low, up)	(−0.018, 0.000)	(−0.031, −0.013)
	*p*-value	0.0653	0.0012
Deep gray matter	∆SWS in m/s	0.021	−0.028
	95%—CI (low, up)	(−0.046, 0.004)	(−0.047, −0.008)
	*p*-value	0.1098	0.0268

Figure 7 shows CV values for 2D and 3D data processing based on all volunteers and as an average of individual CVs from baseline, 1-day, and 1-year measurements for eleven volunteers. CV for the total group and subset group (*n* = 31 and *n* = 11) was similar. CV for 2D processing was markedly lower than for 3D processing with the lowest values measured in WM and highest values in DGM. As shown in Figure 7 and indicated by ICC ≥0.95, very good reproducibility was achieved for both pipelines. ICC 95% confidence intervals obtained in 2D were smaller than in 3D. Mean RAD between repeated measurements indicated better reproducibility for 2D than 3D processing (*p* = 0.004 for WM). Specifically, mean RAD was lowest for 2D WM SWS (0.33 ± 0.32%) and increased for GBT (0.43 ± 0.33%), CGM (0.68 ± 0.46%), and DGM (1.31 ± 1.06%). 3D values for GBT, WM, CGM, and DGM were 1.16 ± 0.71%, 0.96 ± 0.66%, 1.47 ± 0.98%, and 1.34 ± 1.17%, respectively. A summary for CV, ICC and RAD is given in Table 2. The corresponding analysis for penetration rate, PR, presented as Supplementary Material, revealed similarly excellent reproducibility of viscosity-related PR for 2D and 3D reconstruction (e.g., ICC in GBT: 0.94 for 2D and 0.98 for 3D). However, the 3D reconstruction showed lower variability for PR than 2D (e.g., mean RAD in GBT: 0.84 ± 0.74% vs. 1.72 ± 1.22%).

Significant brain softening after 1 year was observed in GBT, WM, CGM, and DGM using 3D reconstruction. In GBT, SWS changed between baseline and 1-year follow-up by −0.011 m/s (95%-CI: [−0.021, −0.001], *p* = 0.037) and between day one and 1-year follow-up by −0.021 (95%-CI: [−0.032, −0.010], *p* = 0.007). This longitudinal decrease in SWS, most likely attributable to aging, was about tenfold higher than obtained from our previously reported cross-sectional analysis in 31 brains. In contrast, no significant change in WM volume was observable after 1 year, whereas BPF was significantly reduced by 1.1 ± 1.0% (*p* = 0.019).

### Dependence of 3D SWS values on number of slices

**FIGURE 8:**
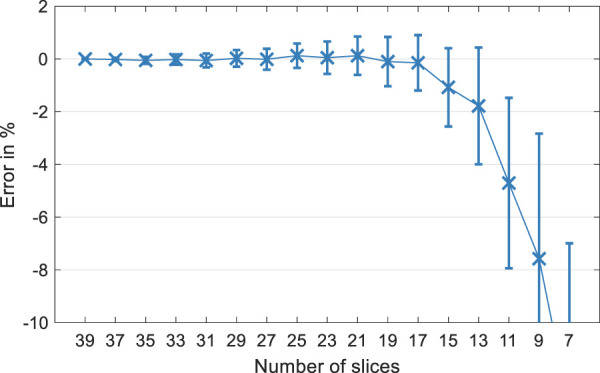
Mean relative error in % for mean white matter SWS using 3D data processing in eleven volunteers. The error is calculated as the relative difference between the reconstructed SWS of the center slice using 39 input slices (reference) and subsequently removing the boundary slices prior to reconstruction.

Figure 8 demonstrates how 3D SWS averaged within WM of the center slice is affected by the total number of input slices for a fixed block thickness. The mean relative error was obtained from eleven volunteers and computed by taking WM 3D SWS of the full 39-slice input data as a reference while subsequently reducing the number of slices by removing the outermost slice pair. For each computation, central-slice SWS was averaged within WM and normalized with reference SWS of the same region. It is apparent that reference SWS is increasingly underestimated (more than 10%) as the total slice number is successively reduced to less than nine slices, indicating the inaccuracy of 3D MRE in thinner slabs.

## Discussion

Introducing cerebral tomoelastography, we address the longstanding challenges of brain MRE, namely high variability of values relative to pathophysiological changes and limited anatomical detail in individual MRE maps. To the best of our knowledge, this is the first study which analyzed brain viscoelasticity changes in healthy volunteers after 1 year. Moreover, we investigated the consistency of MRE parameters by comparing repeated measurement after 1 day and by comparing 2D and 3D data processing. We also performed a correlation analysis with physiological data, to scrutinize the biological and technical margins of reproducibility of the method. In the following, we discuss the results with regard to SWS.

Strikingly, 2D stiffness values were very similar to 3D values (relative differences <5% in WM and <8% GBT), which we consider an important indication of the overall consistency of the proposed inversion pipeline in addition to excellent reproducibility scores.

Exploring brain mechanics in a frequency range between 20 and 35 Hz, we obtained results that are comparable with previously published values from large-scale dispersion analysis (1.22—1.65 m/s for 20–35 Hz) (Herthum et al., 2021b). Converting our GBT SWS values to the magnitude of the complex shear modulus |G*| using the elastic model (2D-|G*| = 1.6 kPa, 3D-|G*| = 1.4 kPa) facilitates a direct comparison with recently reported values obtained at 50 Hz vibration frequency. Hiscox et al. (2016) reported GBT |G*| of 2.07 ± 0.42 kPa and 2.62 ± 0.21 kPa (Hiscox et al., 2020b) while Svensson et al. (2021) reported storage modulus *G’* of 1.29 kPa and 1.76 kPa, depending on the inversion algorithm used. Lv et al. (2020) reported GBT values between 1.6 kPa at 40 Hz and 2.2 kPa at 60 Hz. Yeung et al. (2019) reported WM |G*| values for healthy adults at 30 and 40 Hz driving frequency of 1.13 ± 0.13 kPa and 1.64 ± 0.19 kPa, respectively. Thus, our mean values are in a range that is covered by the body of the MRE literature (Chatelin et al., 2010; Hiscox et al., 2016).

Similarly, our observation of stiffer DGM than WM (10%—20%) and softer CGM than WM (5%—10%) is in agreement with prior research (Hiscox et al., 2020b), with larger contrasts for 3D. Moreover, in 3D reconstruction, the putamen and globus pallidus were stiffer than the nucleus caudate and thalamus, consistent with Hiscox et al. (2020b), while in 2D, only the putamen was markedly stiffer than other regions. Remarkably, our maps from some volunteers show these anatomical subregions with unprecedented detail and in agreement with standard T2-weighted MR images.

Age-related brain softening, as previously shown by different research groups (Sack et al., 2009; Sack et al., 2011; Barnhill et al., 2018; Hiscox et al., 2021), was reproduced in our cross-sectional study. Converted to |G*|, we found an annual decrease in GBT stiffness of 5 Pa (2D) and 7 Pa (3D), consistent with the average decrease of 8 Pa reported by Hiscox et al. (2021). Yet, BPF, which is tightly linked to aging (Peters, 2006), seems to explain most of the age-related changes in GBT SWS. For the first time, we measured annual brain softening due to aging in a test-retest study design. We observed a higher rate of softening when analyzing longitudinal changes with 3D MRE and setting day 0 as reference versus day 1—34 Pa versus 49 Pa. Both values are markedly higher than expected from our cross-sectional design. This difference could have several reasons and deserves further investigation. The younger age range (22–46 years) in our longitudinal study compared with the cross-sectional study (22–61 years) might point towards a more complex process of brain softening than linear reduction in stiffness. Of note, we observed a reduction of BPF over the course of 1 year, but we did not observe a reduction of brain tissue volume or any cross-sectional correlation of brain stiffness with other physiological parameters such as BMI and blood pressure during this period. While we observed a positive correlation between BPF and cross-sectional MRE in 2D and 3D, the longitudinal sensitivity of 3D MRE to WM brain softening suggests that MRE is also sensitive to intrinsic brain tissue changes unrelated to brain tissue loss since WM volume did not change. Our findings suggest that age-related brain softening is markedly smaller than pathology induced changes by e.g., Alzheimer’s disease [approximately 7% (Murphy et al., 2011; Gerischer et al., 2018)]. Nonetheless, age control is becoming increasingly important in cohort studies, especially when MRE technology advances and subtle stiffness changes become detectable. In previous work, we found BMI to be negatively correlated with stiffness in the putamen and globus pallidus (Hetzer et al., 2020), two regions which were not addressed by the hypotheses and correlation analyses of this study.

The consistency of cerebral tomoelastography in terms of within-subject (0.33% RAD, 0.95 ICC) and inter-subject (2% CV) variability is encouraging in comparison with other MRE methods (Murphy et al., 2013; Hiscox et al., 2016; Johnson et al., 2016; Huang et al., 2019) given our small voxel size (<8 mm^3^) and minimal smoothing. It should be pointed out again that the detail resolution of our maps was better than that of most published MRE maps of the brain. Our study has shown that it is not sufficient today to reject methods that provide higher anatomical fidelity than conventional approaches by referring to the lack of ground truth in brain MRE. Ground truth must be defined over many studies, but with consideration of anatomically plausible structures (avoiding hot spots, observing symmetry, delineation of CSF) as well as high consistency of values in follow up examinations, optimally over a year. It is a remarkable result of our study that the consistency of cerebral tomoelastography was similarly good or better as other quantitative MRI techniques (Heiervang et al., 2006; Deh et al., 2015; Gracien et al., 2020). For proton density, T1, T2, and T2* relaxation times of WM, Gracien et al. (2020) reported mean RAD values between 1% and 2% and inter-subject CV values between 2% and 5%. Using diffusion MRI, Heiervang et al. (2006) reported inter-session CV of fractional anisotropy and mean diffusivity below 5% and 3%, while inter-subject CV was below 10% and 8%, respectively. Quantitative susceptibility mapping (Deh et al., 2015) and perfusion imaging were found to have correlation coefficients for repeated measurement of *r* = 0.98, which is considered highly reproducible (Granziera et al., 2021). Thus, cerebral tomoelastography adds an excellently reproducible and biophysics-based imaging marker to these quantitative MRI methods.

It is worth mentioning that a reproducible excitation of wave fields inside the brain and a reproducible selection of imaging volumes are important to achieve a high consistency of MRE values. Our actuator setup is position-sensitive and therefore we ensured that the volunteer’s head was placed in a similar position on the driver setup during all follow-up examinations. Nevertheless, any other actuation setup (Runge et al., 2019; Smith et al., 2020; Qiu et al., 2021; Triolo et al., 2022) that achieves good wave penetration of the brain with high test-retest reproducibility is suitable for tomoelastography and can even further improve the consistency of cerebral MRE. Moreover, the transverse slice blocks were automatically aligned by the scanner. Higher variability with smaller mean values in 3D reconstruction were observed due to noise enhancement by the 3D curl operator and 3D phase gradient calculation, which induces additional through-plane tissue boundary artifacts. Yet, 2D bandpass filtering seemed to blur small regional stiffness differences (e.g., caudate nucleus versus globus pallidus) and small longitudinal changes such as 1-year age effects. Another drawback of 2D MRE is its inability to account for complex wave propagation patterns including through-slice components (Hiscox et al., 2016; Manduca et al., 2021). 3D MRE promised to solve this issue, however, it induced boundary slice artifacts, which corrupted up to several boundary slices in our implementation. In general, it depends on the kernel size used for calculating the finite differences and other preprocessing steps like smoothing and directional filters. Therefore, we cannot fully recommend 3D over 2D MRE, as this study has shown that the influence of boundary slices impoverishes the volume that can be used for unbiased stiffness mapping. For example, we had to exclude eight out of 40 slices, which is a waste of 20% scan time and spatial information. In addition, as shown in Figure 8, a small number of slices for a given slice block thickness affects the numerical stability of 3D *k*-MDEV inversion, confirming that sample points per wavelength and sampled wavelength fraction influence SWS reconstruction, as previously shown by Mura et al. (2020). These findings should be independent of the studied organ. 3D *k*-MDEV may be beneficial for other organs and body regions as well if the spatial support across slices is similar to the in-plane resolution. These technical requirements for 3D MRE should be considered whenever subtle mechanical changes are expected in larger tissue regions, similar to our 1-year follow-up study.

Our study has limitations. First, the number of volunteers in our longitudinal study was rather small, which precluded tests for multiple confounders that may affect brain stiffness over 1 year. Therefore, we focused on the effect of aging within 1 year as the most reproduced and best reported physiological confounder of brain MRE. However, a clear separation of aging effects from loss in BPF was not fully possible. Furthermore, we could not avoid that wave amplitudes varied between individuals even though the technical setup including driver amplitude was identical across all experiments. These amplitude variations are likely due to different head geometries, which affect the efficacy of wave induction. Possibly for that reason, 2D-SWS correlated with wave amplitudes in DGM, which contributed to higher CV values. In the future, variability in brain MRE could be further reduced if wave amplitudes inside the brain were actively controlled using an MRI actuator feedback system. Finally, we focused on frequency compound viscoelasticity maps without in-depth analysis of single frequency data. It would be another interesting and important research question which of the included frequencies in our *k*-MDEV maps contributed most to the variability of our data and how one could further improve the consistency of brain MRE by refinement of the range of vibration frequencies. However, such an analysis would exceed our concise study design. To avoid a lengthy presentation, we confined ourselves to stiffness analysis in the main text while providing the results of viscosity analysis in the Supplementary Material.

In summary, this study introduced cerebral tomoelastography based on 2D and 3D multifrequency MRE and wavenumber-based multifrequency inversion. We assessed reproducibility, long-term changes, detail resolution, and biological effects on viscoelasticity parameters in the healthy human brain. Our method enabled high-resolution viscoelasticity mapping of anatomical detail as demonstrated by the stiffness-based separation of DGM regions in individual volunteers, which was consistent with group mean values. Stiffness correlated with age and BPF whereas BP and BMI did not correlate with MRE values. Cerebral tomoelastography was highly consistent in terms of CV and ICC, 2D versus 3D, and long-term effects. 2D MRE shows less variation across volunteers and at 1-year follow-up than 3D MRE and supports thin imaging slabs while 3D MRE seems to be more sensitive to subtle individual changes such as aging within only 1 year. Overall, cerebral tomoelastography has shown excellent consistency and detail resolution compared with both classical MRE of the brain and other quantitative MRI techniques. Therefore, it contributes to the quest for reproducible, quantitative, and biophysically significant MRI biomarkers for clinical applications.

## Data Availability

The raw data supporting the conclusion of this article will be made available by the authors upon reasonable request.
