# Supplementary material for: Cerebral tomoelastography based on multifrequency MR elastography in two and three dimensions
Source: Front Bioeng Biotechnol. 2022 Dec 2;10:1056131. doi: 10.3389/fbioe.2022.1056131 (PMC9755504; doi:10.3389/fbioe.2022.1056131)
Supplement: Supplementary file 1 [file DataSheet1.pdf]

Supplementary material to

Cerebral tomoelastography based on multifrequency MR elastography in two and three dimensions.

Helge Herthum[1,2,4], Stefan Hetzer[1,2], Bernhard Kreft[3], Heiko Tzschätzsch[3], Mehrgan Shahryari[3], Tom Meyer[3], Steffen Görner[3], Hennes Neubauer[3], Jing Guo[3], Jürgen Braun[4], Ingolf Sack[3*]

[1]Berlin Center for Advanced Neuroimaging, Charité – Universitätsmedizin Berlin, Corporate Member of Freie Universität Berlin, Humboldt-Universität zu Berlin, and Berlin Institute of Health, 10117, Berlin, Germany
[2]Bernstein Center for Computational Neuroscience, Charité – Universitätsmedizin Berlin, Corporate Member of Freie Universität Berlin, Humboldt-Universität zu Berlin, and Berlin Institute of Health, 10117, Berlin, Germany
[3]Department of Radiology, Charité – Universitätsmedizin Berlin, Corporate Member of Freie Universität Berlin, Humboldt-Universität zu Berlin, and Berlin Institute of Health, 10117, Berlin, Germany
[4]Institute of Medical Informatics, Charité – Universitätsmedizin Berlin, Corporate Member of Freie Universität Berlin, Humboldt-Universität zu Berlin, and Berlin Institute of Health, 10117, Berlin, Germany

***Corresponding author:**

Ingolf Sack, PhD
Department of Radiology
Charité – Universitätsmedizin Berlin
Charitéplatz 1
10117 Berlin, Germany
Tel +49 30 450 539058
Ingolf.sack@charite.de

We here present a complementary data analysis for the penetration rate PR reconstructed using the wavenumber-based (*k*-)MDEV inversion.

Supplementary Figure 1 shows three representative slices in MNI space of group averaged PR generated by 2D and 3D processing along with anatomical reference images from the MNI atlas. Masks for WM, CGM and DGM, after the exclusion of cerebrospinal fluid, are demarcated by colored lines. A descriptive summary for all analyzed regions is given in Supplementary Table 1, including region size and CV.

Group statistical plots for GBT, WM, CGM and DGM in 2D and 3D are shown in Supplementary Figure 2. Mean WM PR values were markedly higher for 2D (0.83 ± 0.04 m/s) than 3D processing (0.56 ± 0.03 m/s, $p < 0.0001$). PR was significantly lower in CGM and DGM for 2D reconstruction (CGM: 0.82 ± 0.05 m/s, DGM: 0.77 ± 0.1 m/s, $p < 0.0001$ for each test). For 3D reconstruction, PR was significantly lower in CGM (0.55 ± 0.03 m/s, $p < 0.0001$) and significantly higher in DGM (0.6 ± 0.04 m/s, $p < 0.0001$). A descriptive summary for all analyzed regions is given in Supplementary Table 1, including region size and CV. Intersubject variations as quantified by CV were smaller in 2D than 3D processing. CV in GBT, WM, CGM and DGM was 5.4%, 4.8%, 6.1% and 12.6% in 2D MRE while 4.4%, 3.8%, 5.9% and 6.9% in 3D MRE, respectively (see also Supplementary Figure 6). Supplementary Figure 3 shows a correlation plot for 2D and 3D PR values for GBT. The results for both approaches were highly correlated ($r = 0.74$, $p < 0.0001$).

2D PR was positively correlated with wave amplitudes in the respective region (GBT: $r = 0.74$, $p < 0.0001$, WM: $r = 0.73$, $p < 0.0001$). 3D PR was positively correlated with BPF (GBT: $r = 0.45$, $p = 0.043$) and wave amplitudes in the respective region (GBT: $r = 0.49$, $p = 0.024$, CGM: $r = 0.46$, $p = 0.037$, DGM: $r = 0.65$, $p = 0.0004$).

Supplementary Figure 4 shows the reconstructed PR map in a representative slice from one subject for three measurements: baseline, one day later and after one year for both 2D and 3D processing. No differences between the three measurements were visually apparent. Group averaged PR values (2D and 3D for GBT, WM, CGM, DGM) measured at three time points in eleven subjects are presented in Supplementary Figure 5. Intersubject variability assessed by CV, as well as reproducibility between baseline and one day later assessed by ICC and mean RAD (within subject variability) were derived

from these results and displayed in Supplementary Figure 6. Supplementary Tables 2 summarizes the one-day test-retest and one-year follow-up results, respectively. Supplementary Figure 6 shows CV values for 2D and 3D data processing based on all subjects and as an average of individual CVs from baseline, 1-day and 1-year measurements for eleven subjects. A summary for CV, ICC and RAD is given in Supplementary Table 2. Supplementary Figure 7 demonstrates how 3D PR averaged within WM of the center slice is affected by the total number of input slices for a fixed block thickness.

PR in GBT was roughly 33% lower in 3D than 2D. These differences are likely due to the noise enhancing curl operator which has a stronger effect on the calculation of PR.

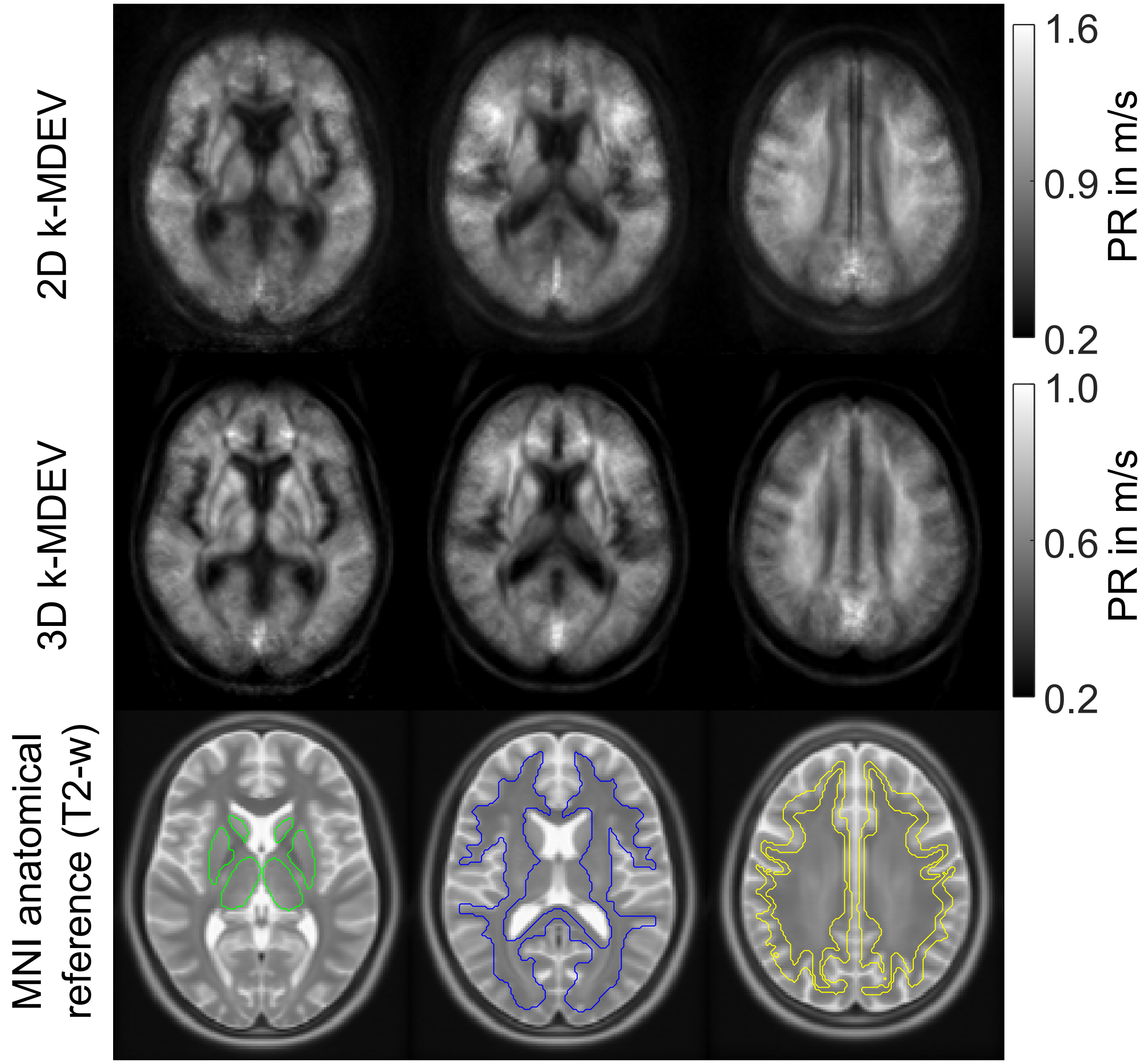

Supplementary Figure 1: Averaged PR maps from 2D and 3D *k*-MDEV inversions normalized to MNI space in three representative slices. Anatomical reference images are shown superimposed with atlas regions for deep gray matter (green), white matter (blue) and cortical gray matter (yellow).

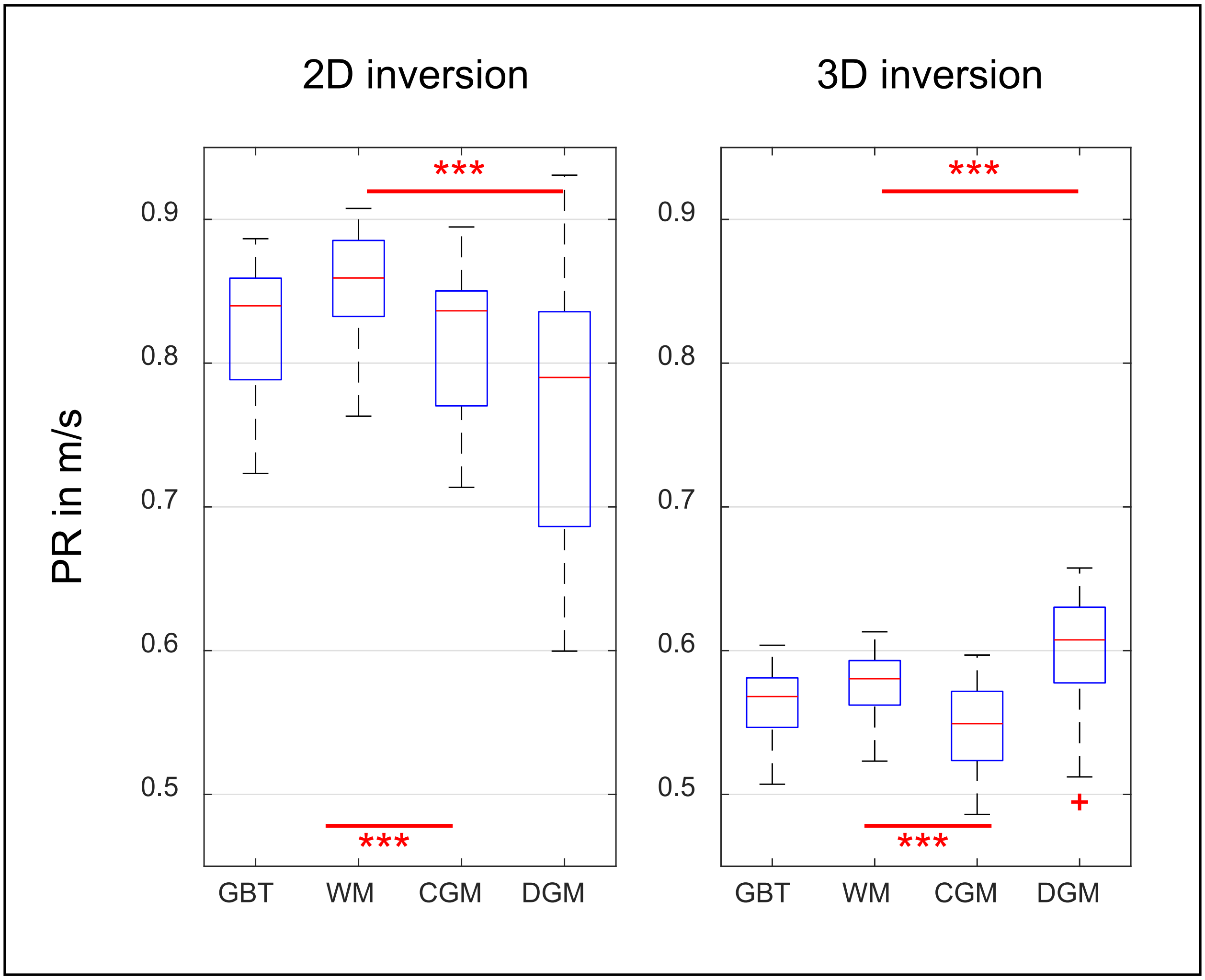

Supplementary Figure 2: Group mean PR values for 2D and 3D *k*-MDEV for global brain tissue (GBT), white matter (WM), cortical gray matter (CGM) and deep gray matter (DGM). Significance levels, indicated by asterisks, were determined from paired t-tests with Holm-Bonferroni correction between WM and CGM as well as WM and DGM.

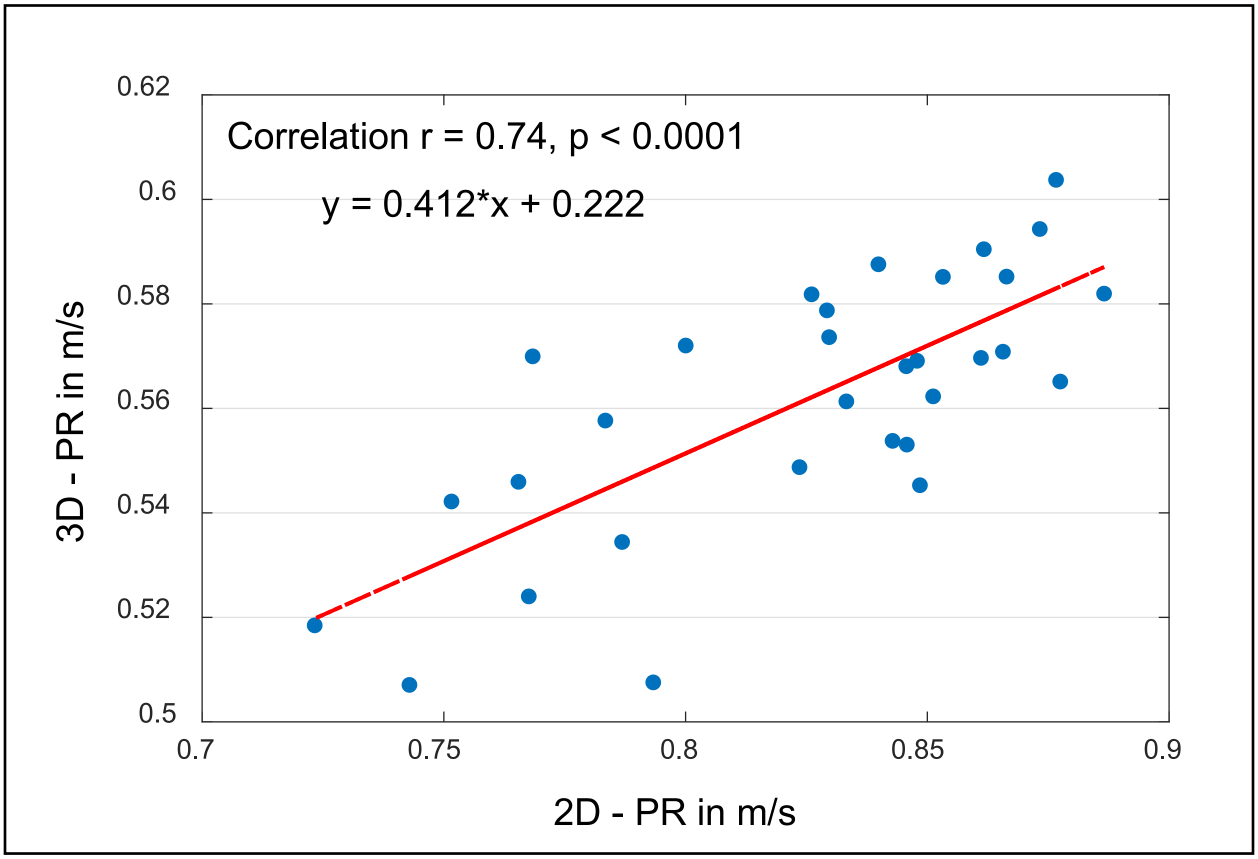

Supplementary Figure 3: Correlation plot for 2D and 3D PR values for global brain tissue.

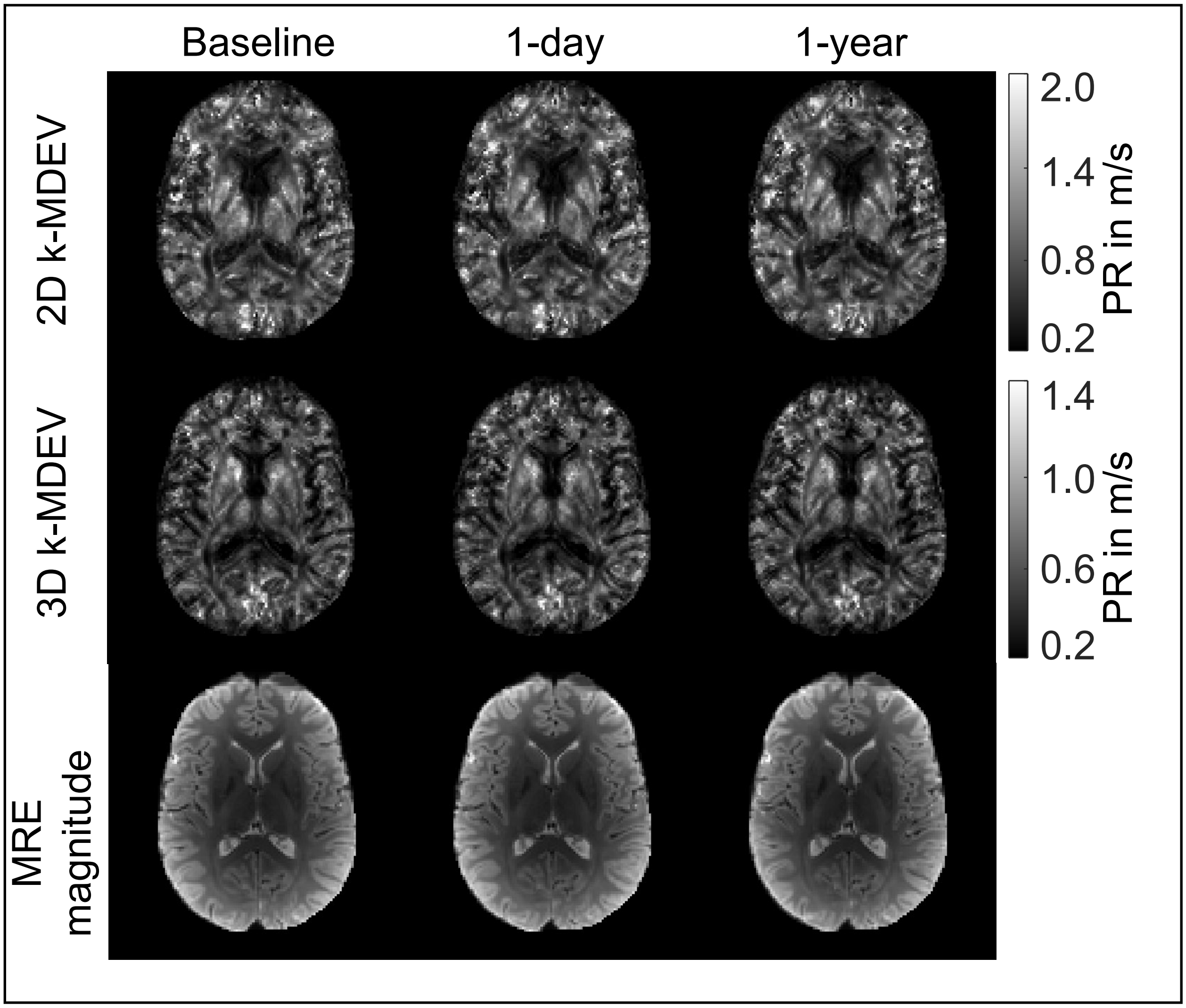

Supplementary Figure 4: Representative MRE penetration rate maps and magnitude images in one subject for three follow-up measurements: baseline, one day later (1-day) and one year later (1-year) for 2D (top) and 3D *k*-MDEV based reconstructions (bottom).

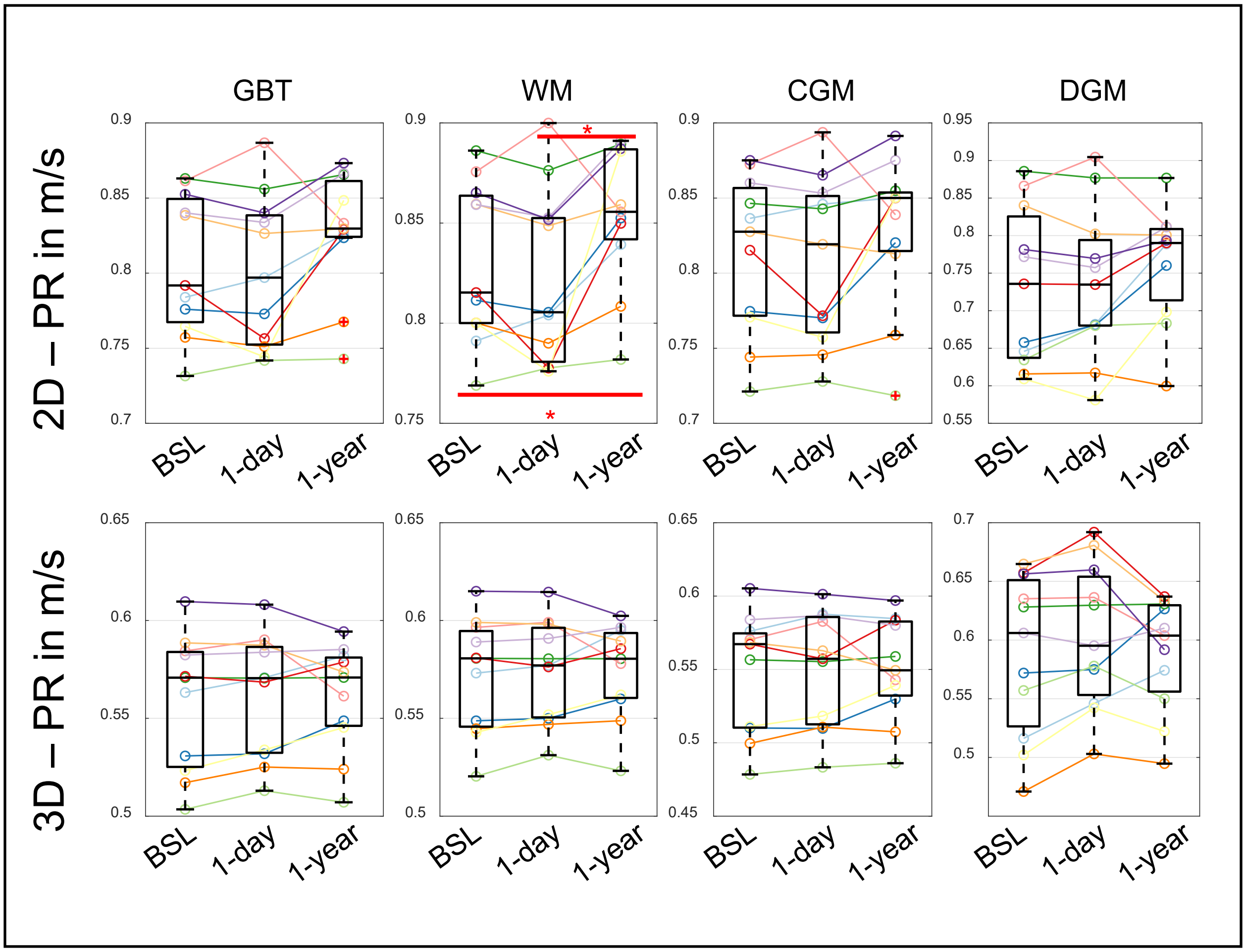

Supplementary Figure 5: Group averaged PR values for 2D (top) and 3D processing (bottom) in global brain tissue (GBT), white matter (WM), cortical gray matter (CGM) and deep gray matter (DGM). Averages were derived from eleven subjects measured at baseline (BSL), one day later (1-day) and after one year (1-year). Significance levels, indicated by asterisks, were determined from paired t-tests with Holm-Bonferroni correction between BSL and 1-year, as well as 1-day and 1-year.

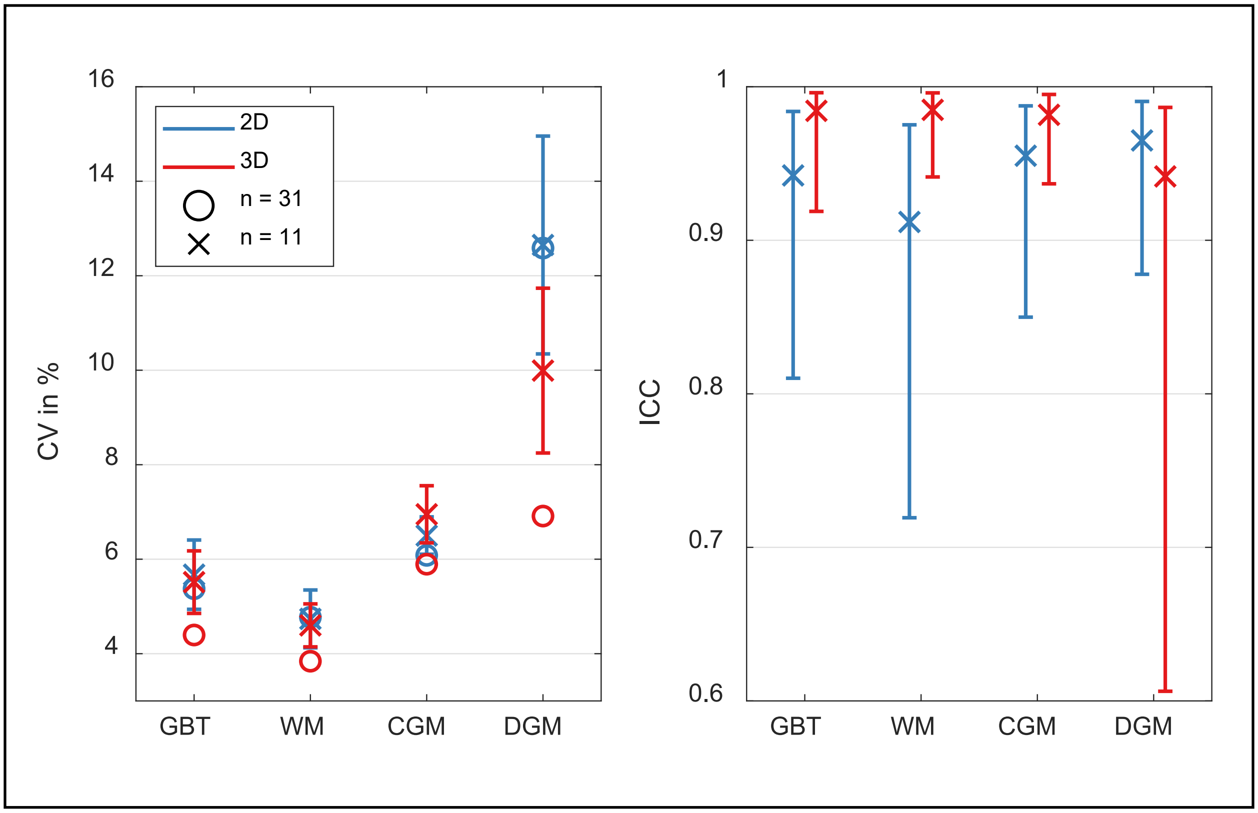

Supplementary Figure 6: Coefficient of variation (CV, left) and intraclass correlation coefficient (ICC, right) for 2D and 3D PR reconstructions for global brain tissue (GBT), white matter (WM), cortical gray matter (CGM) and deep gray matter (DGM). CV determined from single measurement of all subjects and as an average from three CVs for baseline, one day later and after one year repeated tests in eleven subjects. ICC was determined from baseline and after one day repeated measurements.

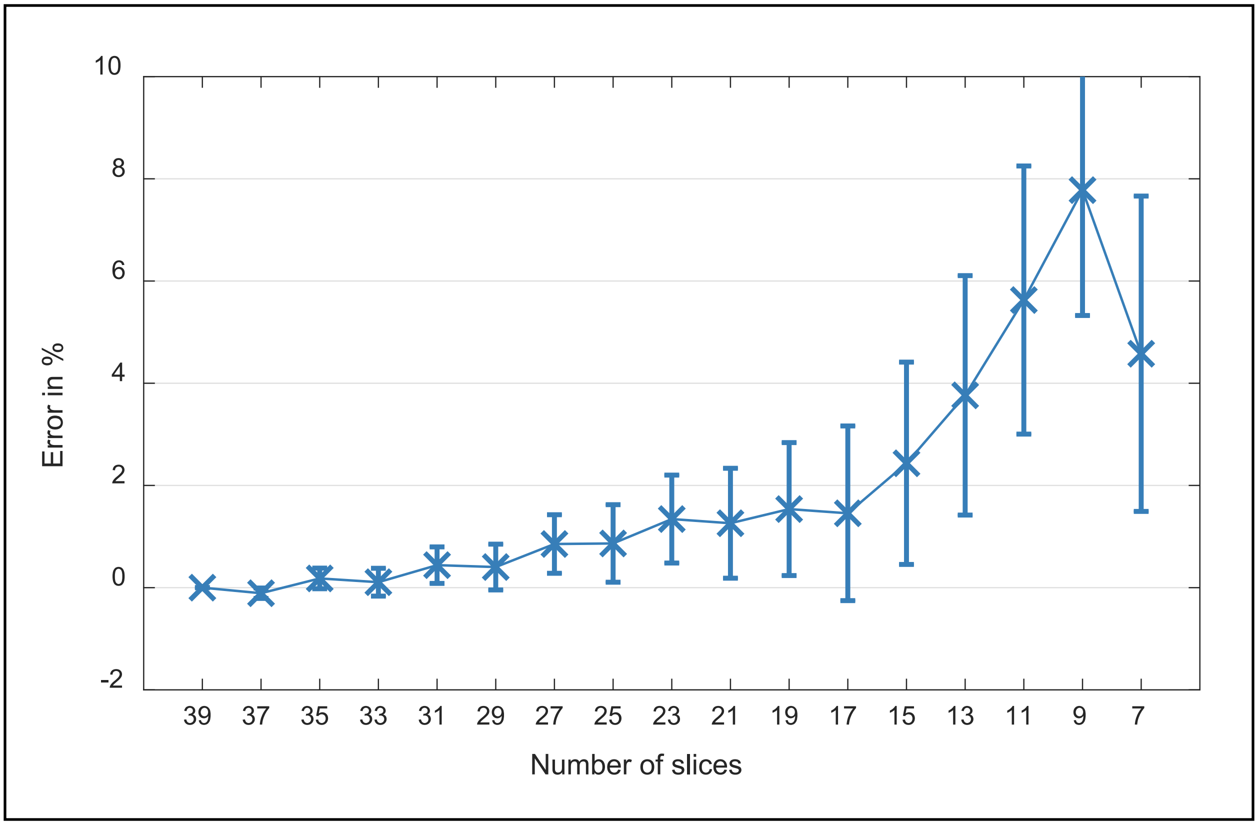

Supplementary Figure 7: Mean relative error in % for eleven subjects for mean white matter PR using 3D data processing. The error is determined by the relative difference between the reconstructed SWS of the center slice using 39 input slices (reference) and subsequently removing the boundary slices prior to the reconstruction.

Tables

Supplementary Table 1: Group mean values for PR for 2D and 3D data processing and the coefficient of variation (CV) for all analyzed brain regions obtained in 31 brains (cross-sectional study): Global brain tissue (GBT), white matter (WM), cortical gray matter (CGM), deep gray matter (DGM), nucleus accumbens (Ac), nucleus caudate (Ca), globus pallidus (Pal), putamen (Pu) and thalamus (Th). Standard deviations are given in brackets. In addition, region size is given.

	2D-PR in m/s	CV in %	3D-PR in m/s	CV in %	Size in cm^3
GBT	0.83 (0.04)	5.4	0.56 (0.03)	4.4	909 (44)
WM	0.85 (0.04)	4.8	0.58 (0.02)	3.8	544 (21)
CGM	0.82 (0.05)	6.1	0.55 (0.03)	5.9	379 (22)
DGM	0.77 (0.1)	12.6	0.6 (0.04)	6.9	53 (5)
Ac	0.7 (0.13)	18	0.68 (0.09)	13.8	1.6 (0.4)
Ca	0.62 (0.13)	20.7	0.57 (0.09)	16.2	10.1 (0.1)
Pal	0.68 (0.11)	16	0.55 (0.07)	12.8	5.5 (0.8)
Pu	0.9 (0.09)	9.5	0.68 (0.05)	7.2	16.5 (1.7)
Th	0.69 (0.12)	17.9	0.5 (0.04)	8.5	26.3 (2.0)

Supplementary Table 2: Coefficient of variation (CV) and intraclass correlation coefficient (ICC) for 2D and 3D PR reconstructions for global brain tissue (GBT), white matter (WM), cortical gray matter (CGM) and deep gray matter (DGM) and DGM subregions. CV is given as an average from three CVs for baseline, 1-day and 1-year measurements for eleven subjects (n = 11). ICC and mean relative absolute difference (RAD) were determined from baseline and 1-day repetition measurements.

2D SWS	mean CV (SD), n = 11	ICC (95%-CI: low, up)	mean RAD (SD, max) in %
GBT	5.67 (0.73)	0.94 (0.81, 0.98)	1.72 (1.22, 4.57)
WM	4.74 (0.61)	0.91 (0.72, 0.98)	1.82 (1.22, 4.74)
CGM	6.50 (0.40)	0.96 (0.85, 0.99)	1.44 (1.48, 5.50)
DGM	12.65 (2.31)	0.97 (0.88, 0.99)	3.11 (2.29, 6.95)
Ac	14.57 (2.57)	0.89 (0.64, 0.97)	4.73 (4.73, 4.92)
Ca	24.96 (4.01)	0.98 (0.92, 0.99)	4.93 (4.93, 2.9)
Pal	16.45 (2.99)	0.94 (0.78, 0.98)	5.49 (5.49, 3.35)
Pu	9.96 (2.2)	0.92 (0.72, 0.98)	4.13 (4.13, 1.87)
Th	17.52 (1.24)	0.98 (0.93, 0.99)	3.16 (3.16, 3.05)
**3D SWS**	**mean CV (SD), n = 11**	**ICC (95%-CI: low, up)**	**mean RAD (SD, max) in %**
GBT	5.51 (0.66)	0.98 (0.92, 1.00)	0.84 (0.74, 2.00)
WM	4.60 (0.46)	0.99 (0.94, 1.00)	0.62 (0.66, 2.06)
CGM	6.95 (0.61)	0.98 (0.94, 1.00)	1.18 (0.76, 2.20)
DGM	9.99 (1.74)	0.94 (0.61, 0.99)	3.12 (2.78, 7.70)
Ac	13.27 (0.92)	0.82 (0.27, 0.95)	6.71 (6.71, 4.41)
Ca	19.91 (1.96)	0.97 (0.91, 0.99)	3.86 (3.86, 2.86)
Pal	16.43 (1.11)	0.94 (0.78, 0.98)	4.93 (4.93, 4.28)

Pu	10.35 (1.21)	0.92 (0.7, 0.98)	3.53 (3.53, 3.18)
Th	9.29 (2.44)	0.94 (0.4, 0.99)	2.93 (2.93, 2.52)
